# New insight into the causal relationship between Graves’ disease liability and drug eruption: a Mendelian randomization study

**DOI:** 10.3389/fimmu.2023.1267814

**Published:** 2023-11-21

**Authors:** Dide Wu, Boyuan Liu, Wei Xian, Yuxin Yang, Jinjian Li, Shubin Hong, Yanbing Li, Haipeng Xiao

**Affiliations:** ^1^Department of Endocrinology, First Affiliated Hospital of Sun Yat-sen University, Guangzhou, Guangdong, China; ^2^Zhongshan School of Medicine, Sun Yat-sen University, Guangzhou, China

**Keywords:** Mendelian randomization study, Graves’ disease, drug eruption, European population, Asian population

## Abstract

**Background:**

Graves’ disease (GD) and drug eruption are closely associated and frequently observed in the clinical setting. However, it remains unclear whether a causal relationship exists between these two conditions. The aim of the study is to investigate whether GD is causal to drug eruptions using two-sample Mendelian randomization.

**Methods:**

We launched a two-sample MR to investigate whether GD is causal to drug eruption using Genome-wide association study (GWAS) summary data from Biobank Japan and FinnGen. Genetic variants were used as instrumental variables to avoid confounding bias. Statistical methods including inverse variance weighted (IVW), weighted median, MR-Egger, and MR-PRESSO were conducted to identify the robustness of the causal effect.

**Results:**

Genetically predicted GD may increase the risk of drug eruption by 30.3% (OR=1.303, 95% CI 1.119-1.516, p<0.001) in the Asian population. In European populations, GD may increase the generalized drug eruption by 15.9% (OR=1.159, 95%CI 0.982-1.367, p=0.080).

**Conclusions:**

We found GD is potentially causal to drug eruption. This finding expanded the view of the frequently observed co-existence of GD and adverse drug reactions involving the skin. The mechanism remains for further investigation.

## Introduction

Graves’ disease (GD) is an autoimmune disease that typically affects the thyroid gland and causes hyperthyroidism (1). The annual incidence of GD worldwide is 20 to 50 cases per 100,000 persons (2). Although GD may occur in anybody, it is more common in women, with a risk of 3% and 0.5% for men (3). The stimulating thyroid-stimulating hormone receptor autoantibodies (TRAbs) are specific and central to GD, and the detection of TRAbs in serum is commonly applied to confirm the diagnosis of GD in clinical practice (1). The histological appearance of the thyroid gland demonstrated classic lymphocytic infiltrate of T cells and B cells, along with typical diffuse follicular cell hyperplasia (4). For the past 70 years, the ‘thioamide’ antithyroid drugs (ATDs) have remained a primary conventional treatment method, along with radioiodine and surgery (5, 6). However, ATDs may induce a series of side effects, among which cutaneous reactions, typically rash, pruritus, and urticaria, represent the predominant adverse effects ascribed to ATDs, with an incidence as high as 6% (6, 7).

Drug eruptions are commonly known as adverse drug reactions involving the skin (8). Adverse cutaneous drug reactions have been recognized as a significant health problem and socioeconomic burden worldwide (9). Meanwhile, up to 2% of all adverse cutaneous drug reactions are considered to be fatal and life-threatening, although most follow a benign course (10). The pathogenesis of severe drug eruptions is complex and may be associated with specific human leukocyte antigens (HLAs) and non-HLA genes, T-cell mediated cytotoxicity (11). It’s been proven that certain autoimmune disorders, such as systemic lupus erythematosus, have a higher incidence of adverse drug eruptions (12). However, it remains unclear whether a causal relationship exists between GD liability and drug eruptions.

Mendelian randomization (MR) is a powerful analytical method that uses genetic variants as instrumental variables (IVs) to generate causal relations between risk factors on outcomes (13). Analogous to the principle of randomized controlled trials (RCTs), the MR study is conducted based on the truth of the random allocation of parental alleles at conception, according to Mendel’s law of inheritance (14). MR findings are less susceptible to confounding and reverse causation since the genetic variants are fixed at conception and cannot be modified thereafter. The principle of MR is based on the gene-environment equivalent assumption. In that condition, modifying the exposure by genetic variation should have the same effect on the outcomes (15). Hierarchically speaking, MR study draws strength from RCT and observational concepts and is located at the interface between traditional observational epidemiology and interventional trials (16).

In the current study, we performed an MR study to investigate whether GD liability is causal to drug eruptions.

## Materials and methods

### Study design

**Figure 1:**
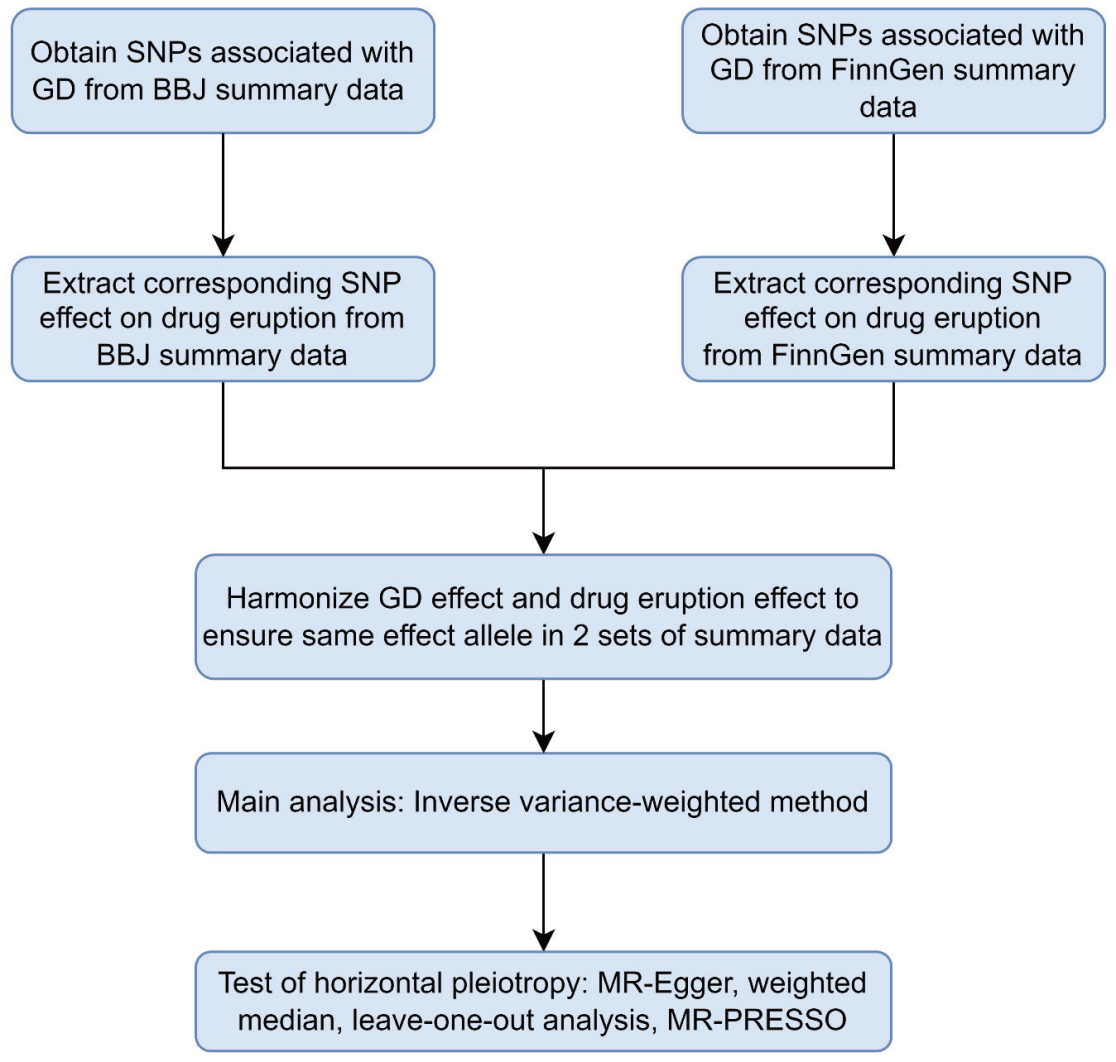
Flowchart of the two-sample Mendelian randomization study for the association between GD and drug eruption. GD, Graves’ disease; SNPs, single-nucleotide polymorphisms; BBJ, Biobank Japan; MR-Egger, Mendelian Randomization Egger; MR-PRESSO, Mendelian Randomization Pleiotropy RESidual Sum and Outlier.

We operated a two-sample MR to investigate the causal relationship between Graves’ disease liability and drug eruption (17). Allele pairs segregate during gamete formation and then distribute to offspring randomly according to Mendel’s laws. This procedure can avoid potential bias by confounders since genetic variants are fixed at fertilization, mimicking an RCT process (16). Qualified genetic variants in Mendelian randomization study should satisfy three fundamental assumptions: (i) the genetic variants should be robustly associated with exposure in interest; (ii) the genetic variants do not influence the potential confounders; (iii) the genetic variants influence the outcome only through the risk factor (13, 18–20). Single nucleotide polymorphisms(SNPs) eligible for MR analysis are called instrumental variables(IVs) (21). We extracted SNPs associated with GD as IVs to investigate the causal effects of GD liability on drug eruption in the East Asian population and European population. We operated different MR analytic methods to ensure IVs satisfy the abovementioned basic assumptions. A flow chart of the analysis process is demonstrated (**Figure 1**). A diagram of the Mendelian randomization method in our study was demonstrated in **Supplementary Figure 1A**. Similarities between MR approach and RCT were demonstrated in **Supplementary Figure 1B**. Ethical approval and patient consent are not required in our research because they had been obtained in previous studies.

### GWAS summary data source

We obtained SNPs associated with GD and drug eruption from GWAS summary data of Biobank Japan (BBJ) and FinnGen, respectively. Biobank Japan(BBJ) is the largest non-European biobank that collects genome and clinical data from more than 200000 individuals of East Asian ancestry (22, 23). BBJ collaboratively collects DNA and serum samples from Japanese patients with a diagnosis of at least one of 47 diseases from 12 medical institutions in Japan (22). The diagnosis of GD and drug eruption was based on physicians’ diagnoses made at cooperating hospitals from BBJ (23). BBJ excluded patients of non-East Asian ancestry or those with a history of bone marrow transplantation (23). GWAS summary statistics from BBJ are publicly available from the JENGER website (http://jenger.riken.jp/en/) and the MRC Integrative Epidemiology Unit Open GWAS database (https://gwas.mrcieu.ac.uk/).

FinnGen consortium collected and analyzed genome and health data from 500,000 Finnish biobank participants (24). We obtained summary data of Graves’ disease(ICD 10 code E05.0), generalized drug eruption (ICD 10 code L27.0), and localized drug eruption (ICD 10 code L27.1)from Freeze 8 of the FinnGen consortium to explore the causal relationship between Graves’ disease liability and drug eruption in the European population. GWAS summary data from FinnGen are publicly available (https://www.finngen.fi/en/access_results).

Descriptive information of data source for Graves’ disease and drug eruption is presented in **Supplementary Table 1**.

### Instrumental variables

SNPs robustly associated with Graves’ disease or drug eruption were selected concerning the first assumption. We extracted SNPs associated with Graves’ disease or drug eruption with P < 5.0 × 10^-8^ to ensure the satisfaction of the relevance assumption of Mendelian randomization (25). β values of the selected SNPs were derived from GWAS study utilizing statistical models like linear or logistic regression. By quantifying the association between a one-unit change in the allele count of the SNP and the studied trait (Graves’ disease), β values play an essential role in estimating the causal relationship between Graves’ disease and the outcome of interest (26).We calculated each SNP’s F statistic (β^2^/SE^2^) and excluded SNPs with F statistics smaller than 10 to avoid weak instrument bias (27). We operated a clumping procedure to ensure independence among SNPs (Clumping window 10000kb, r^2^ threshold 0.01) (17). Linkage disequilibrium (LD) proxy SNP can be used when the target SNP is absent in the outcome summary data (17). We set the threshold of r^2^ at 0.8 to ensure a strong correlation between the target SNP and proxy SNP so that the proxy SNP can replace the target SNP in subsequent MR analysis. We removed palindromic SNPs with effect allele frequencies between 0.3 and 0.7 to avoid ambiguity in identifying the effect allele in the exposure and outcome dataset and confirm the reference strand was reliable (17). We calculated the percentage of variance (R^2^) explained by each SNP based on Lee et al.’s research (28).

### Statistical analysis

We operated multiple MR methods to identify the causal effect of Graves’ disease on drug eruption. We mainly use the random-effects inverse variance weighted method (IVW) to estimate the effect of Graves’ disease liability on drug eruption (17). This method combines the Wald ratio (calculated by β values of exposure and outcome) of individual SNPs and elicits estimates of a causal relationship (29). Specifically, random-effects IVW requires validity of all IVs and balance of horizontal pleiotropy (29). We calculated Cochran’s Q value to assess heterogeneity among IVs (30). We used the weighted median method, MR‒Egger regression method, and leave-one-out analysis to evaluate horizontal pleiotropy (17, 31). The weight-median method requires the validity of only half of the IVs allowing stronger SNPs to contribute more toward the estimate (17, 32). The MR‒Egger method returns an appropriate causal effect even when horizontal pleiotropy presents in IVs included in the analysis (33). The intercept of MR-Egger regression reflects the pleiotropy of SNPs included in our study (19). We operated a leave‐one‐out analysis to avoid bias caused by horizontal pleiotropy from a single SNP by sequentially removing one SNP at a time (17). We applied the Mendelian Randomization Pleiotropy RESidual Sum and Outlier (MR-PRESSO) test to discover potential pleiotropic outliers and adjust MR estimates by removing outliers (34). We operated the Steiger directionality test to confirm the causal direction of Graves’s disease on drug eruption (28, 35). Since the exposure and outcome in both population were derived from the same population, we evaluated the bias of sample overlap based on Burgess et al.’s research (36). We completed the analysis mentioned above by using the TwoSampleMR package (version 0.5.6) in R (version 4.2.1).

## Results

Thirteen SNPs significantly associated with GD in the East Asian population were extracted from the BBJ summary data (**Supplementary Tables 2, 3**). Patients with GD were, on average, 50.7 years old, and patients with drug eruption were, on average, 63.2 years old. Thirteen SNPs associated with Graves’ disease in the European population were identified from FinnGen Freeze 8 summary data (**Supplementary Table 4**). The average age at diagnosis of Graves’ disease, generalized drug eruption, and localized drug eruption is 49.3 years old, 49.3 years old, and 51.4 years old, respectively. Thirteen eligible SNPs associated with Graves’ disease were used to assess the causal effect of Graves’ disease liability on drug eruption in the European population (**Supplementary Tables 5, 6**). Detailed descriptive data of SNPs included in our study are demonstrated in **Supplementary Tables 2–6**.

**Figure 2:**
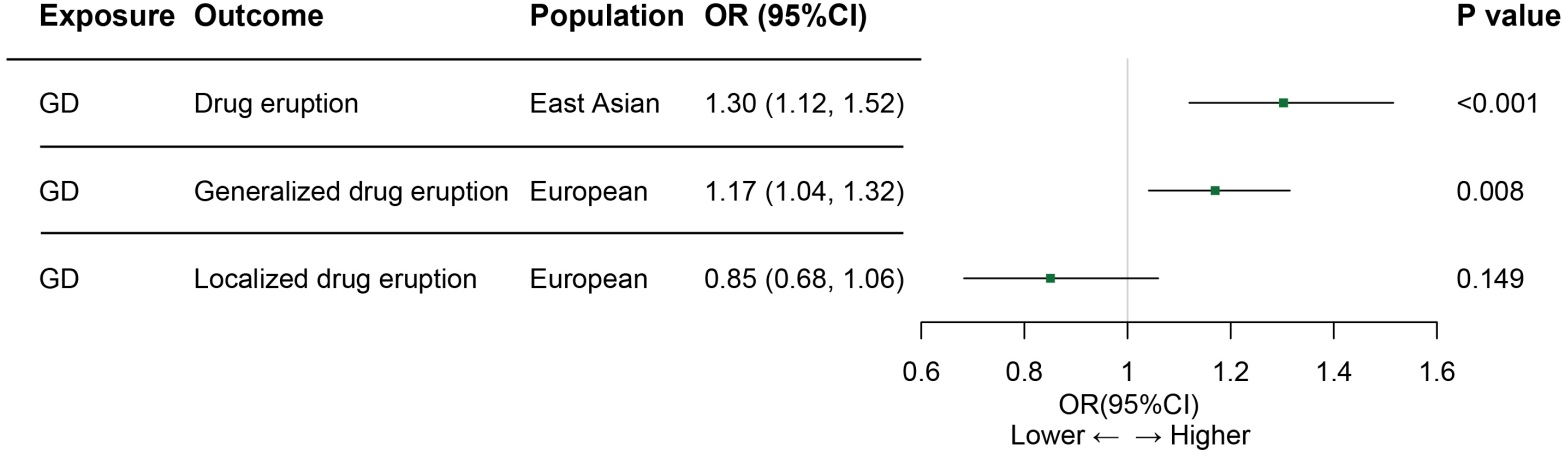
Associations between GD and drug eruption risk in Asian and European populations. GD, Graves’ disease; OR, odds ratio; CI, confidence of interval. Generalized drug eruption, generalized skin eruption due to drugs and medicaments taken internally (ICD 10 code L27.0); Localized drug eruption, localized skin eruption due to drugs and medicaments taken internally(ICD 10 code L27.1).

Results of the MR analysis for both population are summarized in **Figure 2**. In the East Asian population, genetically predicted Graves’ disease liability was causally associated with the development of drug eruption (**Table 1**). We found that genetically predicted Graves’ disease liability may increase the risk of drug eruption by 30.3% in the East Asian population (OR 1.303, 95% CI 1.119-1.516, p<0.001). In other words, we found that the risk of drug eruption increases 8.3% per doubling in odds of GD by multiplying the MR causal estimate by log*_e_* 2 (37, 38).The weighted median method also indicated consistent results (OR 1.380, 95% CI 1.128-1.688, p=0.002). The causal effect contributed by each SNP is demonstrated in the scatter plot and the forest plot (**Supplementary Figures 2A, B**). Horizontal pleiotropy is not detected according to the intercept of MR-Egger regression(p=0.618). Heterogeneity was not presented in included IVs (**Table 1**; **Supplementary Figure 2C**). Leave-one-out sensitivity test showed no single SNP significantly influenced the result of the causal estimate (**Supplementary Figure 2D**).

**Table 1:** MR estimates of the relationship of genetically predicted Graves’ disease on drug eruption.

Exposure	Outcome	Population	Number of SNPs	Method	OR	95% CI	P value	Cochran’s Q (P value)	MR Egger intercept (P value)
GD	DE	East Asian	13	IVW MR Egger Weighted median	1.303 1.484 1.380	1.119-1.516 0.882-2.498 1.128-1.688	<0.001 0.165 0.001	6.915(0.863) 6.652(0.827)	-0.038(0.618)
GD	Generalized DE	European	13	IVW MR Egger Weighted median	1.170 1.066 1.221	1.041-1.315 0.805-1.410 1.042-1.431	0.008 0.664 0.014	12.626(0.397) 12.056(0.359)	0.029(0.486)
GD	Localized DE	European	13	IVW MR Egger Weighted median	0.851 0.722 0.859	0.683-1.060 0.430-1.210 0.633-1.166	0.149 0.242 0.330	8.388(0.754) 7.913(0.721)	0.051(0.505)

GD, Graves’ disease; DE, drug eruption; IVW, inverse variance weighted; OR, odds ratio; CI, confidence interval.

In the European population, genetically predicted Graves’ disease liability may also be relevant to drug eruption (**Table 1**). Genetically predicted Graves’ disease liability may increase the risk of generalized drug eruption (OR 1.170, 95% CI 1.041-1.315, p=0.008). In other words, we found that the risk of generalized drug eruption increases 4.8% per doubling in odds of GD. The weighted median method indicated consistent result(OR 1.221, 95% CI 1.042-1.431, p=0.014). Causal estimates calculated by each SNP are shown in **Supplementary Figures 3A, B**. MR-Egger method indicated that horizontal pleiotropy is not presented(p=0.486). Heterogeneity was not detected in SNPs extracted from FinnGen (**Table 1**; **Supplementary Figure 3C**). No single SNP significantly influences the causal estimate of Graves’ disease on generalized drug eruption (**Supplementary Figure 3D**). However, genetically predicted Graves’ disease liability is not associated with localized drug eruption (OR 0.851, 95% CI 0.683-1.060, p=0.149). Details regarding the association results of Graves’ disease liability and localized drug eruption for each SNP are shown in **Supplementary Figure 4**.

No significant pleiotropic outliers are detected in the analysis of the causal relationship between Graves’ disease liability and drug eruption in the East Asian and European populations considering the MR-PRESSO result (**Supplementary Table 7**). The Steiger directional test confirmed the causal direction of Graves’ disease liability toward drug eruption in both populations (**Supplementary Table 8**). In the evaluation of sample overlap, we found that it did not significantly affect Type 1 errors and did not introduce bias into the results, even with a sample overlap rate of 100%.

## Discussion

To the best of our knowledge, this is the first study investigating the causal relationship between GD liability and drug eruptions using the MR method. Our findings revealed GD itself is an independent causal factor for drug eruptions.

It’s well known that GD may cause dermopathy. GD is a systemic autoimmune disease characterized by thyrotoxicosis caused by circulating stimulatory TRAbs. Primarily, GD affects the thyroid gland with a typical histological appearance of diffused follicular hyperplasia and lymphocyte infiltration (4). Meanwhile, non-thyroidal tissues were also affected secondary to the action of TRAbs and the breakdown of immune tolerance. The most known extrathyroidal manifestations of GD were Graves ophthalmopathy and pretibial myxoedema, altogether known as the Graves Triads (39). GD-associated dermopathy revealed the underlying mechanism that there is a local immune response and subsequent inflammatory reaction embedded in the skin. Although the detailed mechanism of skin involvement in GD is awaiting study, the infiltration of inflammatory cells, including T cells, B cells, dendritic cells, and macrophages with both cytokines and chemokines involvement has been observed across a large spectrum of disease severities (40).

Drug eruption is typically seen as a cutaneous adverse drug reaction. The mechanism is still unclear. Besides the impact of specific drugs that patients received, the common pathogenesis of drug eruption includes genetic susceptibility, such as genetic linkage with HLA and non-HLA genes, T cell receptor restriction, and T cell and neutrophils mediated cytotoxicity mechanisms (8, 41). The association between drug eruptions and HLA alleles has been identified in many pieces of literature, such as HLA-A*31.01, HLA-B*58.01, HLA-B*15.02, HLA-A30, and HLA-B22. Cytokines are involved in the pathogenesis of drug eruptions, including IL-10 (TNF-α/IL-10 imbalance), IL-15, and IL-18 (42). Biomarkers, including eosinophil, miR-18a-5p, miR-124, miR-214, and Th17, are closely related to the activation and development of drug eruptions (43).

For the past 70 years, thionamides have been the most favorable treatment for newly diagnosed GD worldwide (44). Despite their simple molecular structures and ease of use, many remain uncertain, including their mechanism of action and adverse reactions such as drug eruptions. Although minor cutaneous reactions may be sustained by antihistamine therapy as recommended by ATA guidelines, many may result in drug withdrawal and treatment plan switch (6, 45). Drugs with small molecular weight must form complexes with tissue to initiate the pathology process to produce an immune response. Then subsequent hypersensitivity reactions may show as cutaneous changes. Our finding revealed that genetically predicted GD itself might cause drug eruptions. However, the underlying mechanism is awaiting more studies. This may explain the high prevalence of cutaneous reactions in GD patients. Moreover, the complexity of disease-drug-cutaneous reaction may be a drug development target and promote more ideal drugs for the treatment of GD.

There is currently limited research on the potential mechanisms underlying the association between GD and drug eruptions. However, some possible explanations exist for how GD could increase the risk of drug eruptions. Dysregulation of the immune system in GD patients could potentially increase the risk of developing drug reactions by altering the immune response to medications. Meanwhile, increased metabolism can affect how drugs are metabolized and eliminated from the body, potentially leading to drug accumulation or the formation of reactive metabolites that can cause drug eruptions. Moreover, genetic factors may predispose individuals with GD to drug reactions, although this is poorly understood.

There are several limitations of our study. Firstly, our findings provide evidence of a potential causal relationship between GD liability and drug eruption, rather than a direct relationship between GD itself and drug eruption. Rigorous RCTs and further studies are indeed necessary to confirm the direct relationship between GD and drug eruptions. Secondly, this MR analysis mainly used data from an Asian population, and generalizing the results in other populations is warranted. In addition, the underlying pathways remain to be clarified.

## Conclusion

This is the first MR study focusing on the causal relationship between genetically predicted GD and drug eruption. Our study revealed that GD itself might be casual to drug eruption though the underlying mechanism awaits discovery. This finding broadens the understanding of the complex relationships between GD, ATDs, and skin involvement. Clinical awareness of this finding may be essential for improving treatment strategy and avoiding treatment dismal.

## Data availability statement

Publicly available datasets were analyzed in this study. This data can be found here: https://gwas.mrcieu.ac.uk/, https://r8.finngen.fi/.

## Author contributions

DW: Conceptualization, Data curation, Writing – original draft. BL: Data curation, Writing – original draft. WX: Data curation, Writing – original draft. YY: Formal Analysis, Writing – review & editing. JL: Formal Analysis, Writing – review & editing. SH: Formal Analysis, Funding acquisition, Supervision, Writing – review & editing. YL: Conceptualization, Supervision, Writing – review & editing. HX: Conceptualization, Formal Analysis, Project administration, Supervision, Writing – review & editing.

## Supplementary material

Supplementary Figure 1 Diagrams illustrating the Mendelian randomization design in the study of the association between GD and drug eruption. **(A)** Assumptions of Mendelian randomization used in the study. **(B)** Flowchart illustrating the similarities between the process of Mendelian randomization and randomized controlled trial. SNPs, single-nucleotide polymorphisms; GD, Graves’ disease.

Supplementary Figure 2 Visualization of MR result of GD on drug eruption in East Asian population. **(A)** forest plot; **(B)** scatter plot; **(C)** funnel plot; **(D)** leave-one-out result plot; MR, mendelian randomization; GD, Graves’ disease; SNPs, single-nucleotide polymorphisms.

Supplementary Figure 3 Visualization of MR result of GD on generalized drug eruption in European population. **(A)** forest plot; **(B)** scatter plot; **(C)** funnel plot; **(D)** leave-one-out result plot; MR, mendelian randomization; GD, Graves’ disease; SNPs, single-nucleotide polymorphisms.

Supplementary Figure 4 Visualization of MR result of GD on localized drug eruption in European population. **(A)** forest plot; **(B)** scatter plot; **(C)** funnel plot; **(D)** leave-one-out result plot; MR, mendelian randomization; GD, Graves’ disease; SNPs, single-nucleotide polymorphisms.
