# Supplementary material for: New insight into the causal relationship between Graves’ disease liability and drug eruption: a Mendelian randomization study
Source: Front Immunol. 2023 Nov 21;14:1267814. doi: 10.3389/fimmu.2023.1267814 (PMC10703291; doi:10.3389/fimmu.2023.1267814)
Supplement: Supplementary file 1 [file DataSheet_1.zip › SupplementaryTables.DOCX]

Supplementary Table 1. Descriptive details of source of Grave’s disease and drug eruption.

| Phenotype | Case | Control | Ancestry | Consortium | FinnGen Code |
| --- | --- | --- | --- | --- | --- |
| Graves’ disease | 2176 | 210277 | East Asian | Biobank Japan | - |
| Drug eruption | 430 | 209651 | East Asian | Biobank Japan | - |
| Graves’ disease | 2575 | 339924 | European | FinnGen | E4_GRAVES_STRICT |
| Generalized drug eruption | 914 | 341585 | European | FinnGen | GENERALI_SKIN_ERUPT_DRUGS_MEDICAM |
| Localized drug eruption | 245 | 342254 | European | FinnGen | LOCALIZED_SKIN_ERUPT_DRUGS_MEDICAM |

Supplementary Table 2. Details of SNPs associated with Grave’s disease in Biobank Japan(BBJ).

| SNP | Chr | Pos | EA | NEA | EAF | β | SE | P | F statistic | Gene |
| --- | --- | --- | --- | --- | --- | --- | --- | --- | --- | --- |
| rs117201373 | 1 | 160419940 | G | A | 0.0653 | 0.4701 | 0.0646 | 3.29E-13 | 52.96 | . |
| rs11571292 | 2 | 204720139 | A | G | 0.6136 | 0.2447 | 0.0317 | 1.10E-14 | 59.59 | . |
| rs2049218 | 3 | 188122978 | T | C | 0.3886 | -0.1731 | 0.0317 | 4.89E-08 | 29.82 | LPP |
| rs13136820 | 4 | 40307564 | T | C | 0.7193 | -0.2021 | 0.0351 | 8.40E-09 | 33.15 | LINC02265 |
| rs1061537 | 6 | 29937795 | A | G | 0.6236 | -0.3143 | 0.0321 | 1.10E-22 | 95.87 | . |
| rs148781980 | 6 | 32667577 | G | A | 0.1773 | 0.2863 | 0.0408 | 2.32E-12 | 49.24 | . |
| rs9296074 | 6 | 33042598 | G | A | 0.4689 | 0.4415 | 0.0310 | 4.91E-46 | 202.83 | HLA-DPB1, HLA-DPA1 |
| rs4248153 | 6 | 31002527 | G | A | 0.6205 | -0.2673 | 0.0318 | 3.91E-17 | 70.66 | MUC22 |
| rs2456453 | 8 | 128201359 | T | C | 0.3406 | -0.2077 | 0.0348 | 2.35E-09 | 35.62 | CASC19 |
| rs11065783 | 12 | 111396249 | G | A | 0.2695 | 0.2904 | 0.0388 | 7.23E-14 | 56.02 | LOC105369980 |
| rs4903961 | 14 | 81462649 | G | C | 0.6134 | 0.2344 | 0.0319 | 1.96E-13 | 53.99 | TSHR |
| rs9319588 | 16 | 30930983 | T | C | 0.9055 | -0.3047 | 0.0531 | 9.63E-09 | 32.93 | FBXL19-AS1 |
| rs1569723 | 20 | 44742064 | A | C | 0.6152 | 0.1857 | 0.0316 | 4.06E-09 | 34.53 | . |

Abbreviations: Chr, Chromosome; SNP, single nucleotide polymorphism; EA, effect allele; NEA, non-effect allele; EAF, effect allele frequency; SE, standard error;

Supplementary Table 3. Details of SNPs associated with Grave’s disease and association with drug eruption in Biobank Japan(BBJ).

| SNP | Chr | Pos | EA | NEA | β | SE | EAF | P |
| --- | --- | --- | --- | --- | --- | --- | --- | --- |
| rs117201373 | 1 | 160419940 | G | A | 0.0747 | 0.1377 | 0.0653 | 0.587 |
| rs11571292 | 2 | 204720139 | A | G | -0.0351 | 0.0700 | 0.6137 | 0.616 |
| rs2049218 | 3 | 188122978 | T | C | -0.0867 | 0.0702 | 0.3886 | 0.217 |
| rs13136820 | 4 | 40307564 | T | C | -0.0186 | 0.0776 | 0.7193 | 0.810 |
| rs1061537 | 6 | 29937795 | A | G | -0.1227 | 0.0705 | 0.6234 | 0.082 |
| rs148781980 | 6 | 32667577 | G | A | 0.0099 | 0.0894 | 0.1776 | 0.912 |
| rs9296074 | 6 | 33042598 | G | A | 0.1791 | 0.0681 | 0.4690 | 0.009 |
| rs4248153 | 6 | 31002527 | G | A | -0.0431 | 0.0702 | 0.6203 | 0.539 |
| rs2456453 | 8 | 128201359 | T | C | -0.1338 | 0.0769 | 0.3405 | 0.082 |
| rs11065783 | 12 | 111396249 | G | A | 0.1255 | 0.0847 | 0.2695 | 0.138 |
| rs4903961 | 14 | 81462649 | G | C | -0.0074 | 0.0705 | 0.6133 | 0.916 |
| rs9319588 | 16 | 30930983 | T | C | -0.0717 | 0.1163 | 0.9054 | 0.538 |
| rs1569723 | 20 | 44742064 | A | C | 0.0511 | 0.0699 | 0.6153 | 0.465 |

Abbreviations: Chr, Chromosome; SNP, single nucleotide polymorphism; EA, effect allele; NEA, non-effect allele; EAF, effect allele frequency; SE, standard error;

Supplementary Table 4. Details of SNPs associated with Grave’s disease in FinnGen.

| SNP | Chr | Pos | EA | NEA | EAF | β | SE | P | F statistic | Gene |
| --- | --- | --- | --- | --- | --- | --- | --- | --- | --- | --- |
| rs2476601 | 1 | 113834946 | G | A | 0.851995 | -0.25925 | 0.036772 | 1.79E-12 | 49.70369 | PTPN22 |
| rs3087243 | 2 | 203874196 | A | G | 0.330288 | -0.25107 | 0.030291 | 1.15E-16 | 68.70126 | CTLA4 |
| rs1794277 | 6 | 32700886 | A | G | 0.100766 | 0.714013 | 0.037459 | 5.32E-81 | 363.325 | . |
| rs2281389 | 6 | 33092019 | G | A | 0.162236 | -0.22399 | 0.039746 | 1.74E-08 | 31.75951 | . |
| rs72891915 | 6 | 33508423 | A | G | 0.039961 | 0.470968 | 0.061639 | 2.16E-14 | 58.3812 | . |
| rs16869677 | 6 | 33908305 | T | C | 0.077919 | 0.310268 | 0.047322 | 5.51E-11 | 42.98896 | LOC |
| rs61734579 | 6 | 35317942 | C | A | 0.038653 | -0.54317 | 0.086449 | 3.32E-10 | 39.47704 | DEF6 |
| rs11038350 | 11 | 45200412 | G | C | 0.307219 | 0.204738 | 0.029446 | 3.57E-12 | 48.34518 | PRDM11 |
| rs179255 | 14 | 80971258 | A | G | 0.591388 | -0.29913 | 0.027841 | 6.31E-27 | 115.4367 | TSHR |
| rs17767904 | 16 | 79716078 | A | G | 0.310133 | 0.204109 | 0.029578 | 5.18E-12 | 47.61906 | LOC |
| rs1569723 | 20 | 46113425 | A | C | 0.728201 | 0.182399 | 0.032299 | 1.63E-08 | 31.89149 | . |
| rs12482947 | 21 | 42431928 | C | T | 0.579147 | 0.166839 | 0.028644 | 5.73E-09 | 33.92491 | UBASH3A |
| rs1985790 | 22 | 22712936 | A | G | 0.458269 | -0.1941 | 0.028912 | 1.90E-11 | 45.06905 | LOC |

Abbreviations: Chr, Chromosome; SNP, single nucleotide polymorphism; EA, effect allele; NEA, non-effect allele; EAF, effect allele frequency; SE, standard error.

Supplementary Table 5. Details of SNPs associated with Grave’s disease and association with generalized drug eruption in FinnGen.

| SNP | Chr | Pos | EA | NEA | EAF | β | SE | P |
| --- | --- | --- | --- | --- | --- | --- | --- | --- |
| rs2476601 | 1 | 113834946 | G | A | 0.8520 | -0.0903 | 0.0655 | 0.1679 |
| rs3087243 | 2 | 203874196 | A | G | 0.3303 | 0.0295 | 0.0499 | 0.5548 |
| rs1794277 | 6 | 32700886 | A | G | 0.1008 | 0.1543 | 0.0783 | 0.0487 |
| rs2281389 | 6 | 33092019 | G | A | 0.1622 | 0.0082 | 0.0635 | 0.8971 |
| rs72891915 | 6 | 33508423 | A | G | 0.0400 | -0.0897 | 0.1210 | 0.4584 |
| rs16869677 | 6 | 33908305 | T | C | 0.0779 | 0.1747 | 0.0876 | 0.0461 |
| rs61734579 | 6 | 35317942 | C | A | 0.0387 | 0.1017 | 0.1209 | 0.4003 |
| rs11038350 | 11 | 45200412 | G | C | 0.3072 | 0.0053 | 0.0508 | 0.9165 |
| rs179255 | 14 | 80971258 | A | G | 0.5914 | -0.0567 | 0.0479 | 0.2364 |
| rs17767904 | 16 | 79716078 | A | G | 0.3101 | 0.0395 | 0.0511 | 0.4391 |
| rs1569723 | 20 | 46113425 | A | C | 0.7282 | 0.0831 | 0.0528 | 0.1154 |
| rs12482947 | 21 | 42431928 | C | T | 0.5791 | 0.0876 | 0.0477 | 0.0661 |
| rs1985790 | 22 | 22712936 | A | G | 0.4583 | -0.0395 | 0.0484 | 0.4154 |

Abbreviations: Chr, Chromosome; SNP, single nucleotide polymorphism; EA, effect allele; NEA, non-effect allele; EAF, effect allele frequency; SE, standard error;

Supplementary Table 6. Details of SNPs associated with Grave’s disease and association with localized drug eruption in FinnGen.

| SNP | Chr | Pos | EA | NEA | EAF | β | SE | P |
| --- | --- | --- | --- | --- | --- | --- | --- | --- |
| rs2476601 | 1 | 113834946 | G | A | 0.8520 | 0.0385 | 0.1274 | 0.7624 |
| rs3087243 | 2 | 203874196 | A | G | 0.3303 | 0.0206 | 0.0963 | 0.8310 |
| rs1794277 | 6 | 32700886 | A | G | 0.1008 | -0.1125 | 0.1513 | 0.4571 |
| rs2281389 | 6 | 33092019 | G | A | 0.1622 | 0.1694 | 0.1224 | 0.1664 |
| rs72891915 | 6 | 33508423 | A | G | 0.0400 | -0.4271 | 0.2308 | 0.0642 |
| rs16869677 | 6 | 33908305 | T | C | 0.0779 | -0.037 | 0.1687 | 0.8263 |
| rs61734579 | 6 | 35317942 | C | A | 0.0387 | 0.2750 | 0.2347 | 0.2413 |
| rs11038350 | 11 | 45200412 | G | C | 0.3072 | -0.0089 | 0.0979 | 0.9275 |
| rs179255 | 14 | 80971258 | A | G | 0.5914 | -0.0725 | 0.0924 | 0.4328 |
| rs17767904 | 16 | 79716078 | A | G | 0.3101 | 0.0137 | 0.0984 | 0.8890 |
| rs1569723 | 20 | 46113425 | A | C | 0.7282 | -0.0541 | 0.1020 | 0.5957 |
| rs12482947 | 21 | 42431928 | C | T | 0.5791 | 0.0904 | 0.0920 | 0.3256 |
| rs1985790 | 22 | 22712936 | A | G | 0.4583 | 0.0983 | 0.0926 | 0.2887 |

Abbreviations: Chr, Chromosome; SNP, single nucleotide polymorphism; EA, effect allele; NEA, non-effect allele; EAF, effect allele frequency; SE, standard error.

Supplementary Table 7. MR-PRESSO results of the causal relationship between genetically predicted Graves’ disease and drug eruption.

| Exposure | Outcome | RSSobs | Global *p* | Number of Outliers |
| --- | --- | --- | --- | --- |
| GD ^a^ | DE ^a^ | 8.457 | 0.876 | 0 |
| GD ^b^ | Generalized DE ^b^ | 14.422 | 0.435 | 0 |
| GD ^b^ | Localized DE ^b^ | 9.665 | 0.756 | 0 |

^a^ Data from Biobank Japan

^b^ Data from FinnGen

Abbreviations: GD, Graves’ disease; DE, Drug eruption; MR-PRESSO, Mendelian randomization pleiotropy residual sum and outlier; RSSobs, observed residual sum of squares.

Supplementary Table 8. Steiger test results of the causal direction from genetically predicted Graves’ disease to drug eruption.

| Exposure | Outcome | R^2^.exp | R^2^.out | Steiger P | Causal direction |
| --- | --- | --- | --- | --- | --- |
| GD ^a^ | DE ^a^ | 0.060 | 0.007 | <0.001 | TRUE |
| GD ^b^ | Generalized DE ^b^ | 0.050 | 0.003 | <0.001 | TRUE |

^a^ Data from Biobank Japan

^b^ Data from FinnGen

Abbreviation: R^2^.exp: Proportion of variance of exposure explained by included instrumental variables; R^2^.out: Proportion of variance of outcome explained by included instrumental variables.
