# Supplementary figures and images for: New insight into the causal relationship between Graves’ disease liability and drug eruption: a Mendelian randomization study
Source: Front Immunol. 2023 Nov 21;14:1267814. doi: 10.3389/fimmu.2023.1267814 (PMC10703291; doi:10.3389/fimmu.2023.1267814)

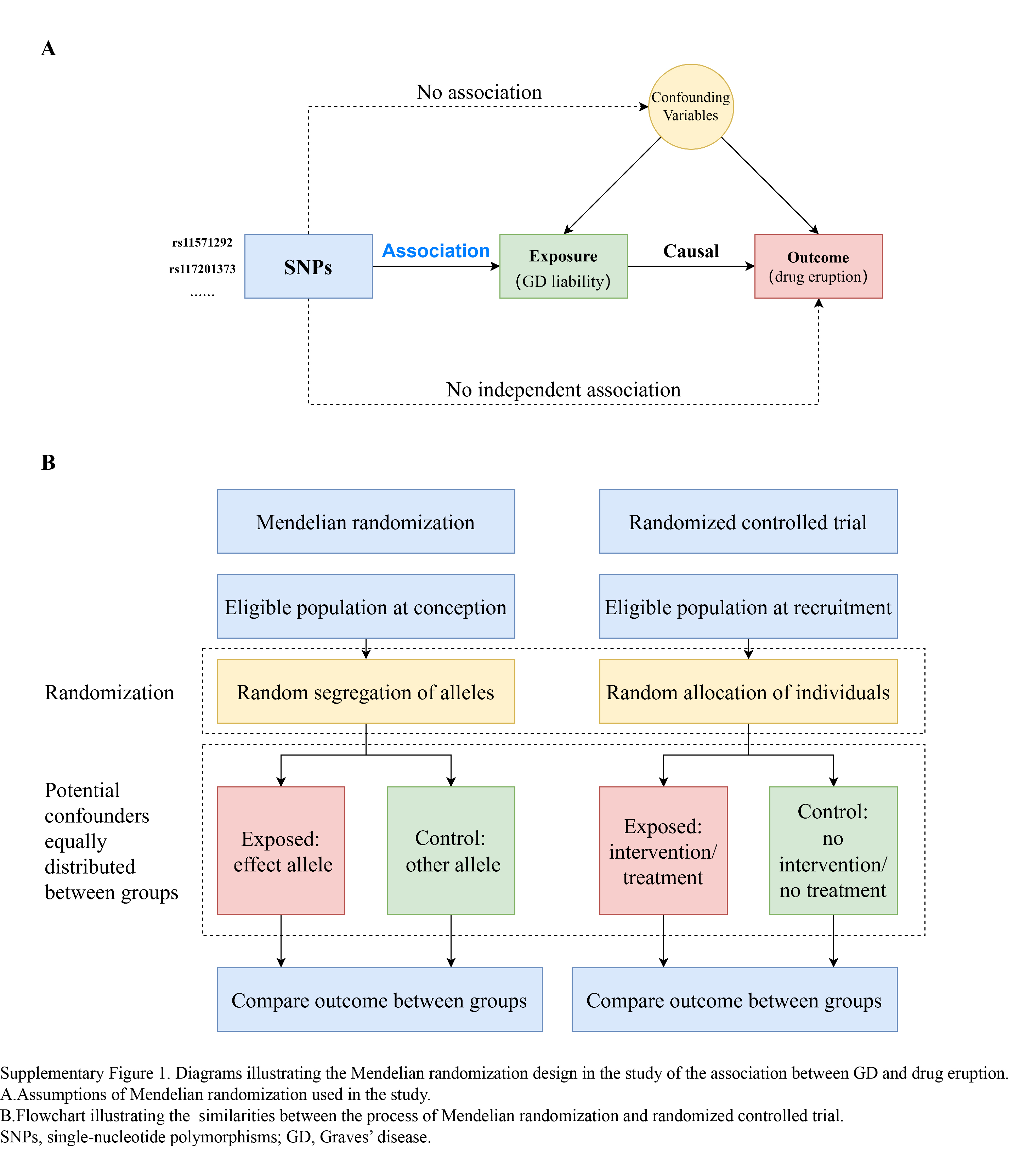

Supplement: Supplementary file 1 [file DataSheet_1.zip › SupplementaryFigure1.jpg]

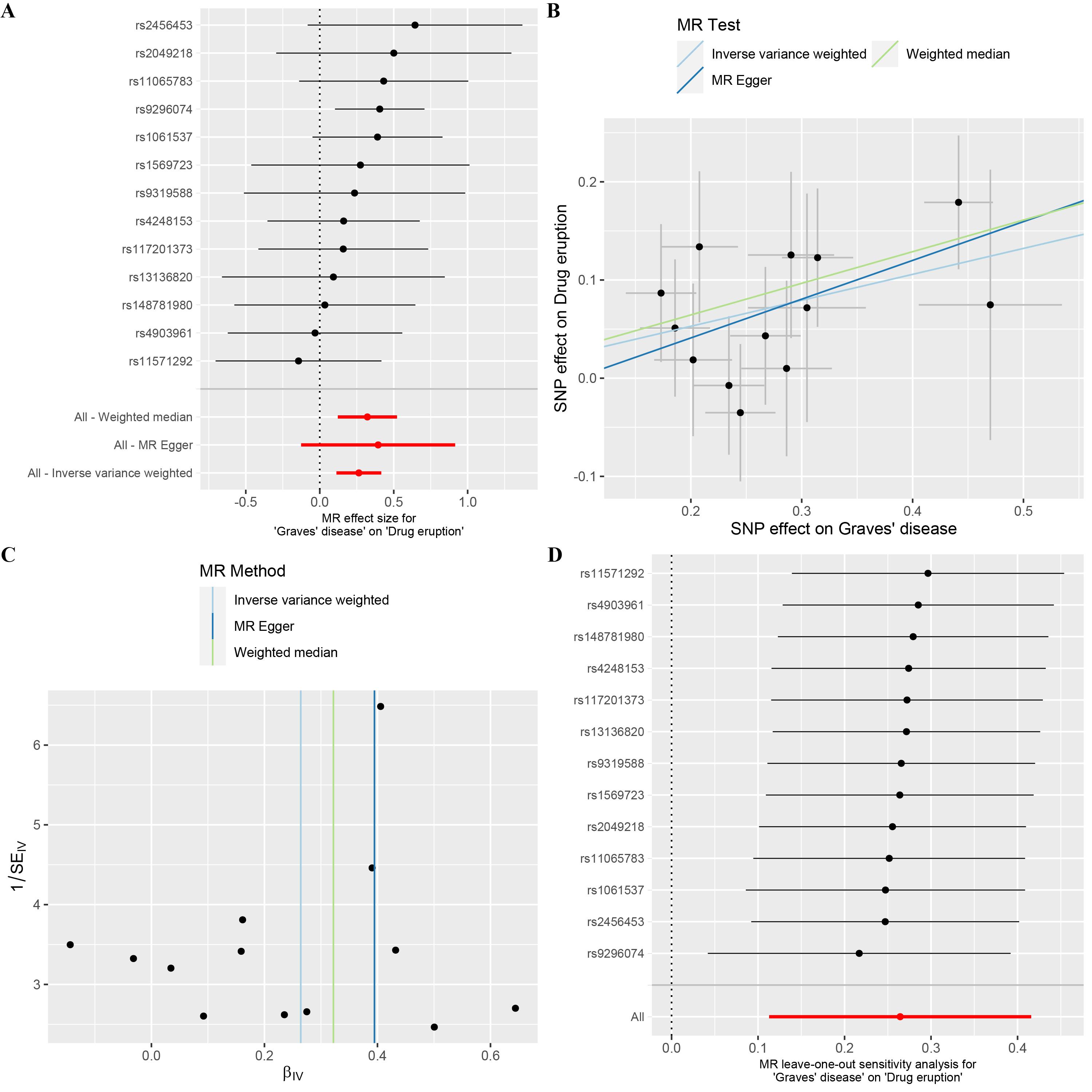

Supplement: Supplementary file 1 [file DataSheet_1.zip › SupplementaryFigure2.jpg]

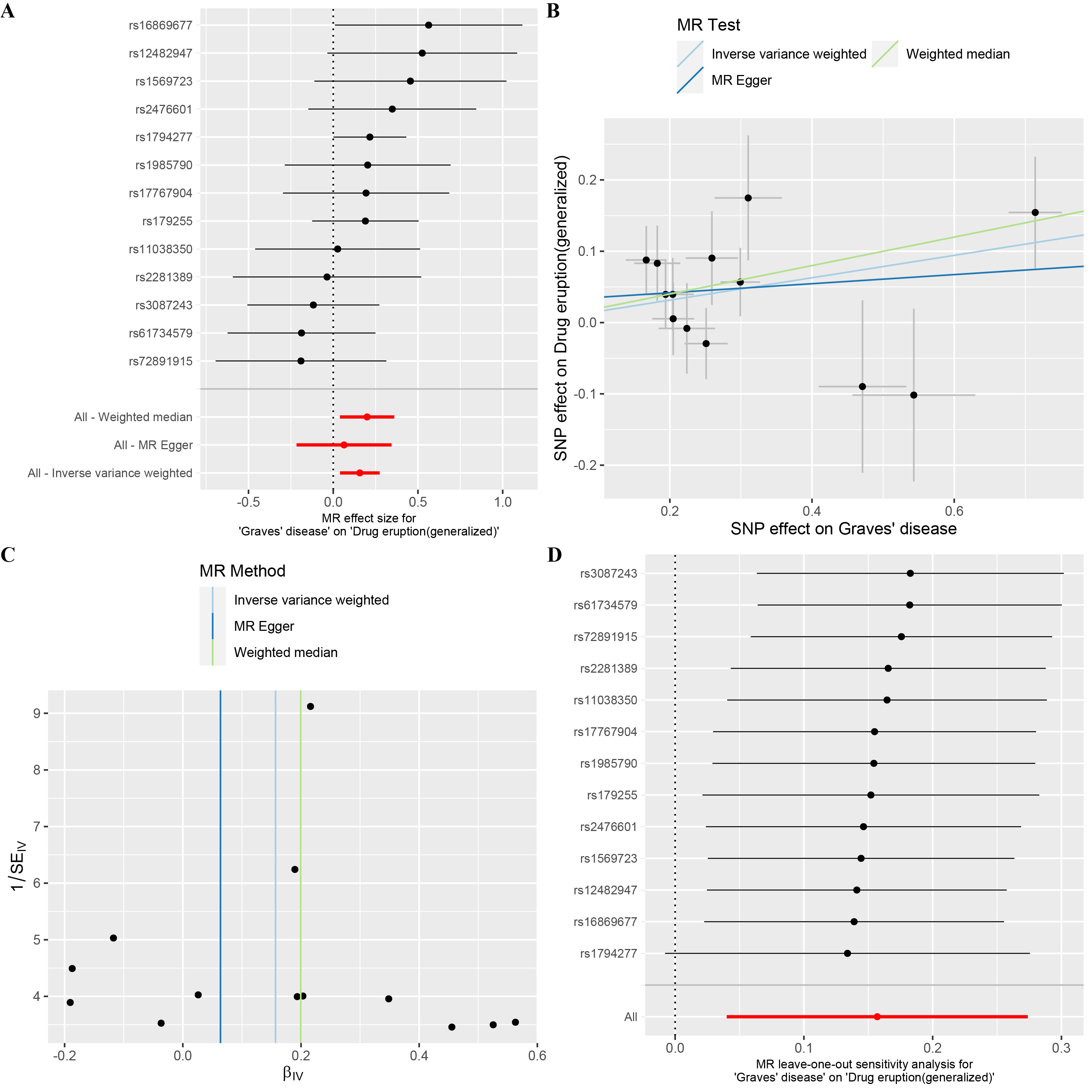

Supplement: Supplementary file 1 [file DataSheet_1.zip › SupplementaryFigure3.jpg]

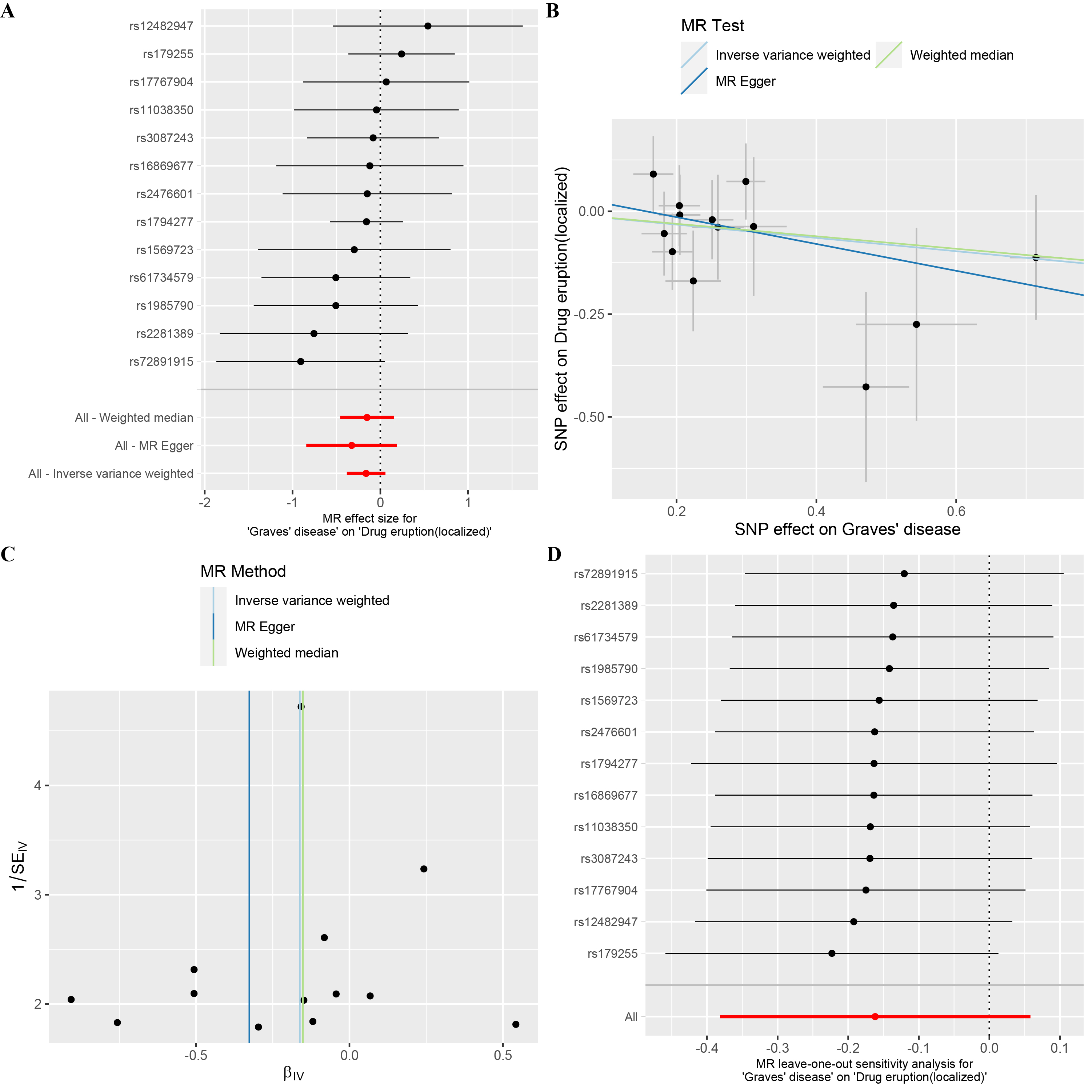

Supplement: Supplementary file 1 [file DataSheet_1.zip › SupplementaryFigure4.jpg]
